# The Genetic and Cellular Basis of Autosomal Dominant Polycystic Kidney Disease—A Primer for Clinicians

**DOI:** 10.3389/fped.2017.00279

**Published:** 2017-12-18

**Authors:** Adrián Cordido, Lara Besada-Cerecedo, Miguel A. García-González

**Affiliations:** ^1^Grupo de Genética y Biología del Desarrollo de las Enfermedades Renales, Laboratorio de Nefrología (n.° 11), Instituto de Investigación Sanitaria (IDIS), Complexo Hospitalario de Santiago de Compostela (CHUS), Santiago de Compostela, Spain

**Keywords:** autosomal dominant polycystic kidney disease, genetics, molecular biology, diagnosis, therapy

## Abstract

Autosomal dominant polycystic kidney disease (ADPKD) is one of the most common genetic disorders worldwide. In recent decades, the field has undergone a revolution, starting with the identification of causal ADPKD genes, including *PKD1, PKD2*, and the recently identified *GANAB*. In addition, advances defining the genetic mechanisms, protein localization and function, and the identification of numerous pathways involved in the disease process, have contributed to a better understanding of this illness. Together, this has led to a better prognosis, diagnosis, and treatment in clinical practice. In this mini review, we summarize and discuss new insights about the molecular mechanisms underlying ADPKD, including its genetics, protein function, and cellular pathways.

## Introduction

Polycystic kidney disease (PKD) is a heterogeneous group of monogenic disorders characterized by the bilateral formation and progressive expansion of renal cyst that lead to end stage renal disease (ESRD) (1). Several Mendelian diseases including autosomal dominant polycystic kidney disease (ADPKD), autosomal recessive polycystic kidney disease (ARPKD), and atypical forms of PKD can be grouped under this pathological entity.

Autosomal dominant polycystic kidney disease is the most common inherited kidney disease affecting ~1/400–1/1,000 individuals (2). The hallmark characteristic of ADPKD is the progressive development and expansion of cysts in the kidney leading to ESRD. It can be associated with several extrarenal manifestations including hypertension, symptomatic extrarenal cysts, and subarachnoid hemorrhage from intracranial aneurysms (3–5). The vast majority of the patients develop the disease between the ages of 20–40 s, but there have been sporadic cases that range in onset from late to childhood (“early onset,” before 15 years old) or even *in utero* (“very early onset”) (6).

## Genetics of the ADPKD

**Figure 1:**
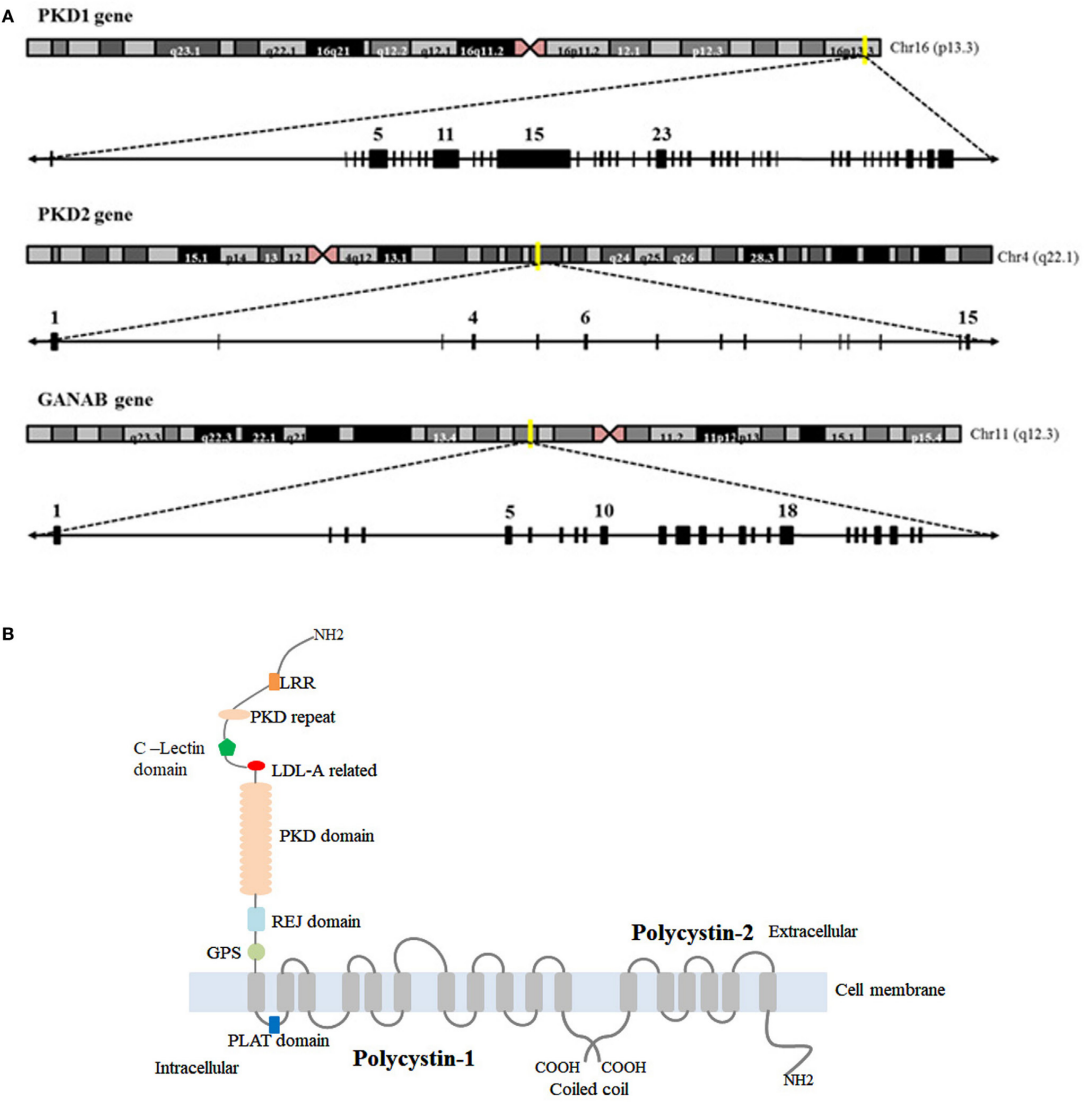
Chromosome localization and genomic structure of *PKD1, PKD2*, and *GANA*β genes and structure of polycystin-1 (PC1) and polycystin-2 (PC2). **(A)** Schematic representation of chromosomes and genomic structure for the genes. **(B)** Representation of *PKD1* and *PKD2* protein products: PC1 and PC2. *GIIα (encoded by GANAβ) is not included because the tertiary structure of the protein is not available in the bibliography or protein date bases.

Autosomal dominant polycystic kidney disease is genetically heterogeneous and associated with mutations in *PKD1* (responsible of ADPKD-Type I), *PKD2* (-Type II), and *GANAB*. *PKD1* is a complex gene mapping to chromosome 16 (16p13.3) (Figure 1A). Its genomic structure has a number of features that complicate its evaluation (7, 8): (a) it is highly GC-rich with a large number of CpG dinucleotides, (b) 70% of *PKD1* is duplicated multiple times throughout chromosome 16 with high sequence fidelity (95% identity) (9), and (c) it contains a 2.5 kb polypyrimidine tract in intron 21 (the largest in the human genome) (10). In contrast to *PKD1*, the *PKD2* gene is located on chromosome 4 (4q21) and has simpler features and structure (11) (Figure 1A). Approximately, 80–85% of ADPKD families were associated with *PKD1* mutations, and 15–20% to *PKD2* mutations in the literature (12). Recently, Porath and colleagues identified causal mutations in *GANAB*, a gene on chromosome 11q12.3 (Figure 1A), in ADPKD patients that are negative for *PKD1* and *PKD2* mutations. They report that *GANAB* accounts for ~0.3% of total ADPKD and it is associated with a milder manifestation of PKD and autosomal dominant polycystic liver disease (ADPLD) (5).

## Diagnosis of ADPKD

The diagnosis of ADPKD is dependent on the stage of the disease. When the disease is fully established, the diagnosis is clinically based on patient’s history and physical examination (13, 14). However, definitive diagnosis can be difficult due to other disorders having overlapping symptoms. Therefore, complementary approaches such as diagnostic imaging or genetic tests are necessary to confirm the diagnosis. Imaging techniques, including ultrasound, computed axial tomography, or nuclear magnetic resonance, allow for the detection of cysts in the kidney, liver, or pancreas (15). The magnetic resonance technique has proven to be more sensitive than ultrasound, allowing measurements of height-adjusted total kidney volume (htTKV) and better definition of the cysts without the use of contrast agents. However, these imaging tests are expensive (16) and are often not performed on a subset of the ADPKD population including those who are young individuals at risk or patients with atypical or *de novo* renal cystic disease (13) for whom complementary genetic tests is the method for definitive diagnosis. Direct DNA sequencing (DS) could offer a molecular diagnosis; however, the genetic analysis of the *PKD1* (responsible for most ADPKD cases) is complicated. The 5′-region of the gene (exon 1–34) is replicated in at least six highly homologous copies on chromosome 16 (7, 9, 17). To date, direct sequencing based on a Long-Rage PCR strategy with specific primers has been the accepted strategy by the ADPKD community (18). Isolated gene by gene sequencing is laborious and expensive, and provides limited amount of information to provide a better diagnosis and prognosis for the patients. Moreover, it has been described that the main mutation responsible of the disease may interact with other PKD or ciliopathy loci modifying the phenotype and extending the genetic complexity of the disease (18–20). For this reason, ADPKD experts are highlighting the necessity to screen all cystic genes in a common strategy to allow for a more accurate diagnosis, including those genes responsible of atypical forms of PKD. Under this context, next-generation sequencing strategies followed by the validation of variants by DS have become the recommended methodology allowing for faster, more cost-effective, and more reliable genetic diagnosis of large ADPKD cohorts (21).

## Genotype–Phenotype Correlations

It has been described that patients with mutations in *PKD1* gene have larger kidneys and earlier onset (mean age at ESRD, 53.4 versus 72.7 years old, respectively) with lower eGFR and higher htTKV than *PKD2* patients (22, 23). In addition, *GANAB* mutations seem to be associated with a mild renal phenotype, closer to a *PKD2* than a *PKD1* phenotype, revealing the importance of molecular diagnosis (5). Moreover, a strong correlation between the type of the mutation and the severity of the disease was observed, illustrating the importance of quantifying genetic heterogeneity in ADPKD. Truncating *PKD1* mutations (frameshift, splicing, and nonsense) have a more severe disease prognosis with lower eGFR; however. the type of mutation does not correlate with htTKV (23, 24). Non-truncating *PKD1* mutations (missense, inframe deletion/insertion) or mutations in *PKD2* are associated with a milder form of the disease. In addition, males with truncating *PKD1* are associated with larger kidneys and increased risk for ESRD, while women with truncating *PKD1* have a more severe liver phenotype (23, 24). In addition, disease manifestation in ADPKD patients from the same family, or patients with the same mutation, can have varying severity and differential disease progression, which may be due to the presence of variation in a modifying gene. This phenomenon is known as genetic interaction and epistasis, and usually aggravates or attenuates the phenotype cause by the primary mutation (17, 25).

Based on genetic and clinical data, Cornec-Le Gall and colleagues (26) developed a robust prognostic model, the PROPKD score (with a range from 0 to 9), to predict survival in ADPKD patients. They described critical variables associated with ESRD including age of onset (median age reported to be 70.6 years for low risk, 56.9 years for intermediate risk, and 49 years for high risk) and a scoring system to predict disease progression: sex (being male 1 point), need for antihypertensive therapy before 35 years old (2 points), occurrence of the first urologic event before 35 years old (2 points), and genetic status (having PKD2 mutations: 0 points, non-truncating PKD1 mutation: 2 points and truncating PKD1 mutations: 4 points). Three risk categories were then defined to describe the putative risk for progression to ESRD: low risk (0–3 points), intermediate risk (4–6 points), and high risk (7–9 points) (26).

## PKD Proteins: Structure and Function

*PKD1* and *PKD2* encode the proteins PC1/Polycystin-1 and PC2/Polycystin-2 or TRPP2, respectively. PC1 is a putative receptor for an unidentified ligand which contains a long extracellular N-terminal domain, 11 transmembrane domains and a short intracellular C-terminal domain (27). PC2/TRPP2 has similar characteristics to TRP channel, having six transmembrane segments, a pore loop domain (separating the first two transmembrane segments), and an N- and C-terminal domains (28) (Figure 1B). PC2 is a Ca^2+^-permeable non-selective ion cation channel and together with PC1 forms a receptor–channel complex implicated in the Ca^2+^ pathway called PC complex (29).

In contrast to *PKD1* and *PKD2, GANAB* encodes the alpha subunit of glucosidase II (GIIα) which is the catalytic subunit of GII. GIIα together with the regulatory subunit of GII, GIIβ (also called hepatocystin) (5) form a functional holoenzyme in the endoplasmic reticulum (ER). This holoenzyme is implicated in the proper folding and translocation of glycoproteins into the ER, and its dysfunction has been reported to be associated with maturation and localization defects of PC1 (30).

## Disease Mechanism

### Two-Hit Model for ADPKD

The human kidney has approximately one million nephrons, and an ADPKD patient will develop around a 1,000 cysts (31). ADPKD disease progression is highly variable and depends directly from the nature of the mutated gene. The “Two-Hit Model,” in which two different mutations affect proper genetic/cellular interactions, has been the proposed theory to explain the kidney phenotype observed in ADPKD patients. While an individual has inherited a germ line mutation (“first hit”), the development of cysts does not occur until another mutation (somatic mutations) in either *PKD1* or *PKD2* occurs (“second hit”) (32, 33).

### Localization of PKD Proteins: The Role of Primary Cilia

There have been a number of different localizations proposed for the PC1/PC2, including the ER, apical and basolateral cell membranes, or secreted exosomes (31). However, there is evidence supporting their presence in primary cilia based on their central role in ADPKD pathogenesis. Cilia are microtubule-based, non-motile organelles on the apical surface of the cells and play an essential role in cellular detection and regulation of external signals. Dysfunction of this organelle result in a group of disorders called the ciliopathies (34). Data from animal models (*C. elegans, Drosophila*, and *Mus musculus*) support the idea that defects in function or structure of primary cilia contribute to the pathomechanisms of PKD (35). PKD proteins such as PC1, PC2, and polyductin/FPC (encoded from the ARPKD gene, *PKHD1*) localized to the primary cilium (36–38). These PKD proteins interact with each other (17, 29, 36, 39, 40) and form a functional complex with common downstream signaling pathways (41). In addition, deleted in azoospermia interacting protein 1-like, the protein encoded from the recently identified ARPKD gene (*DZIP1L*), was reported to localize to the centrioles and basal bodies of cilia and are also associated with ciliary trafficking defects (19).

There has also been additional evidence to support the functional role of PKD proteins within the cilium. Urine flow has been linked to an increase in intracellular calcium (42), likely driven by the mechanical response of the primary cilium (43). The large extracellular domain of PC1 has been proposed to be the flow mechanosensor that opens the PC2-channel, allowing calcium entry leading to mechanotransduction activation (44). A different model proposes that the primary cilia’s role in flow sensing is required for proper centrosomal localization that results in oriented cell division (OCD). In addition, defects in cilia drive the loss of planar cell polarity and consequently abnormal OCD (45); however, this model is controversial and remains unclear (46, 47). Several observations support the idea of the mechanosensory role of polycystins in the primary cilium (48, 49). PC2 directly interacts with KIF3A and KIF3B, two essential proteins for ciliary assembly and function (37, 50). In addition, PC2 is required for the flow-mediated increase of cytosolic Ca^2+^ (51, 52), and mechanical stimuli can induce proteolytic cleavage of the intracellular C-terminal domain of PC1 (53). Interestingly, there have been controversial results reporting that mechanosensation does not occur *via* Ca^2+^ signaling within cilia (54). In spite of this, there are some unanswered questions as while Delling and colleagues do not exclude the presence of others mechanosensitive elements in primary cilia (55) and the cilia seems to increase cytoplasmic Ca^2+^ concentration by diffusion (56).

A very interesting and unexpected finding by Ma and colleagues showed that loss of cilia results in a significant reduction of PKD severity (57). Authors reported that a simultaneous inactivation of polycystins and cilia assembly resulted in the reduction of the cystic phenotype associated with polycystins inactivation. These findings suggest that the polycystins modulate a pathway involved in the cilia signaling, but require intact cilia function (58).

### Threshold or Dosage Model

Genetic background influences the phenotypic variability of ADPKD. As we previously mentioned, patients with mutations in *PKD1* have worse prognosis than those with mutations in *PKD2* (59), and those with truncating *PKD1* mutations were associated with more severe polycystic renal pathology than those with non-truncating mutations (60). In addition, unaffected patients who carry a missense variant in *PKD1* indicate that some alleles are incompletely dominant in the disease (61). Similarly, other studies suggest that incomplete, penetrant alleles can influences disease severity in ADPKD (62).

These data support that a threshold or dosage model could explain cystogenesis in ADPKD (63). According to this model, cyst initiation and cystic expansion depends on PKD gene dosage, starting when the level of functional PC falls below the cystogenic threshold (58, 63). Defects in that threshold may occur by a combination of one or more factors: the nature of germline mutation (“first-hit”), somatic mutations (“second hit”), modifier genes or environmental factors such as renal injury or inflammation (58, 64). Several studies support this: (1) García-González and colleagues reported genetic interaction between ADPKD and ARPKD genes in a common pathway (17), (2) it has been reported that ADPLD genes (*Prkcsh* and *Sec63*), ARPKD gene (*Pkhd1*) and ADPKD gene (*Pkd1*) interact with each other suggesting a central role of PC1 in cystogenesis (65), and (3) a developmental window for cystogenesis has been identified, suggesting that timing of secondary events may influence the severity of ADPKD (66).

**Figure 2:**
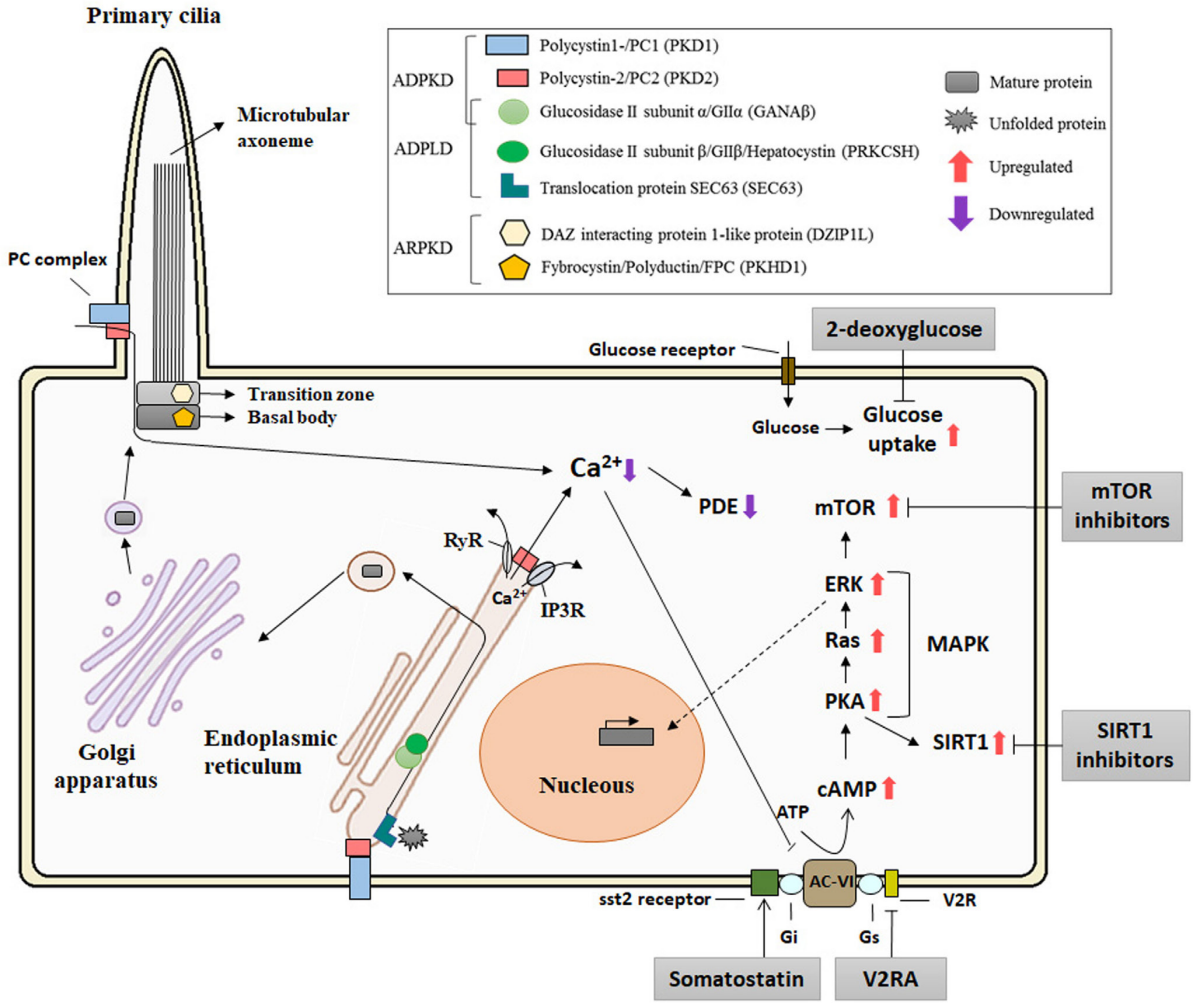
Diagram of the localization of the polycystic kidney disease (PKD) proteins, the pathways implicated in its pathogenesis, and putative therapeutic targets. The polycystin-1 (PC1) and polycystin-2 (PC2), associated with autosomal dominant polycystic kidney disease (ADPKD), and FPC and DIZP1L, associated with autosomal recessive polycystic kidney disease (ARPKD), are ciliary proteins and have functions in relation with the primary cilia. GANAβ [associated with ADPKD and autosomal dominant polycystic liver disease (ADPLD)], together with the classical genes of the ADPLD, PRKCSH, and Sec63, is localized in the endoplasmic reticulum (ER) and plays a role in the translocation and folding of the protein maturation. PC1 and PC2 form a receptor–channel complex in the cilium and is implicated in the Ca^2+^ pathway. PC2 also regulates intracellular calcium in the ER. PC mutations result in deregulation of Ca^2+^ leading an increase in cAMP and upregulation of the PKA and MAPK pathways. Abbreviations: RyR, ryanodine receptor; IP3R, IP3 receptor; PDE, phosphodiesterase; AC-VI, adenylyl cyclase 6; Gs and Gi, guanosine nucleotide-binding proteins; V2R, V2 receptor; cAMP, cyclic AMP; PKA, protein kinase A; MAPK, MAP kinases; SIRT1, sirtuin 1.

A crucial step in the protein maturation of functional PCs is also related to the *dosage model*. Autoproteolytic cleavage of PC1 at the GPS domain, mediated by larger GAIN [G protein-coupled receptor (GPCR)-autoproteolysis inducing] domain which includes a GPCR proteolysis site (GPF) motif, is crucial for PC1 maturation (67, 68). Besse and colleagues have described that specific isolated-PLD proteins (encoded by SEC61β, ALG8, and GANAβ), from the ER protein biogenesis pathway, are directly related to PC1 biogenesis (30). Furthermore, PC1 maturation requires PC2 in a dose-dependent manner (69). It is also known that mature PC assembles at the PC complex-bearing vesicles in the Golgi before trafficking to the ciliary/plasmatic membrane (70) (Figure 2). In addition, Cai and colleagues described the effect of several mutations in *Pkd1* and *Pkd2* in the importance of PCs trafficking to cilia using *in vitro* and *in vivo* models, concluding that altered trafficking and dysfunctional maturation of PC complex underlie PKD pathology (71) These facts suggest a central role for PC1 in the cystogenesis process and in regulating the severity of ADPKD, ARPKD, and ADPLD (72).

### Signaling Pathways and Targeted Therapies in ADPKD

Several signaling pathways and transcription factors control the progression and development of cystogenesis (64, 73). Calcium signaling is one of the most studied pathways in the PKD field. PC2 is a calcium permeable non-selective cation channel that is abundantly expressed in the ER and interacts with others calcium channel proteins (51). It binds to the inositol 1,4,5-trisphosphate receptor (IP_3_R) regulating Ca^2+^ homeostasis and the activity of ryanodine receptors (74, 75). In contrast to PC2, PC1 accelerates the decay of the intracellular calcium response to ATP by increasing ER calcium uptake. All of this suggests a major role of polycystins in intracellular calcium hometostasis (76, 77). Cystic epithelial cells have an aberrant cross talk between intracellular calcium and cAMP signaling as elevated levels of cAMP stimulate cyst fluid secretion, enhancing protein kinase A activity (78–80). Furthermore, V2 receptor antagonists (Tolvaptan) ameliorate the progression of PKD by the inhibition of cAMP signaling pathway in both animal models (81, 82) and in clinical trials (83) (Figure 2). Importantly, adverse secondary effects could appear with Tolvaptan treatment such as polyuria, nocturia, and elevation of liver enzymes (84). Taking this into account, Tolvaptan is the first therapy approved for indication of ADPKD in several countries.

Alterations to other pathways have been reported to affect cystic volume or cystic progression, but to date; attempts to completely inhibit cystogenesis have been unsuccessful. The mTOR pathway is highly activated in cystic tissues independent of the PKD gene mutation (85). Preclinical trials with sirolimus and everolimus blocked cystic progression in a rodent model of ADPKD (86, 87). In addition, somatostatin analogs, as octreotide and lanreotide reduced hepatic and renal volume expansion in ADPKD (88–90). Treatment of PKD animal models, which have defective glucose metabolism associated with cystic expansion, with 2-deoxyglucose, an analog of glucose, also result in reduced cystic progression (91, 92) (Figure 2). Adding to the hunt for therapeutics, other alternate mechanisms also exist, such as sirtuin 1, microRNAs, and MCP1 which have been postulated as possible therapies for PKD (93–95) and recently, ongoing Phase-II and Phase-I clinical trials of a multi-kinase inhibitor, tesevatinib, are ongoing for ADPKD and children with ARPKD (96, 97).

## Conclusion

In the last two decades, there have been significant contributions toward our understanding of the genetic, cellular, and functional role of PKD genes and proteins, as well as, the identification of a number of pathways implicated in the pathogenesis of the disease. Nevertheless, several questions remain unresolved and controversial in the PKD community. The complexity of this disease is reflected along all scientific levels, starting at the genetic level with critical refinement of the mutations, followed by the study of protein function and dosage to understand the spectrum of clinical manifestations in PKD, and finally the study of related pathways and modifier mechanisms that all should be taken into account for future clinical trials and personalized medicine.

## Author Contributions

All authors listed have made a substantial, direct, and intellectual contribution to the work and approved it for publication.

## Conflict of Interest Statement

The authors declare that the research was conducted in the absence of any commercial or financial relationships that could be construed as a potential conflict of interest.
